# Advances in Nanotechnology-Assisted Delivery of TCM-Derived Bioactive Compounds for Wound Repair

**DOI:** 10.3390/pharmaceutics18040427

**Published:** 2026-03-30

**Authors:** Lu Ren, Zefeng Zhao, Tianzihan Zhang, Meiting Kou, Xiaozhen Ma, Jiajun Li, Mengchen Lei, Haifa Qiao

**Affiliations:** 1 School of Basic Medicine, Shannxi University of Chinese Medicine, Xianyang 712046, China; 2111112@sntcm.edu.cn (L.R.);; 2 Institute for Chinese Medicine Frontier Interdisciplinary Science and Technology, Shannxi University of Chinese Medicine, Xianyang 712046, China; 3 School of Clinical Medicine, Shannxi University of Chinese Medicine, Xianyang 712046, China

**Keywords:** Traditional Chinese Medicine, wound healing, multi-target therapy, herbal active ingredients, nanodelivery system

## Abstract

Healing skin wounds is still difficult in many clinical situations, especially when the wounds are chronic or infected. These wounds often stay inflamed for long periods, and the risk of bacterial invasion is high. Oxidative stress tends to increase as well, while the formation of new blood vessels is often inadequate. Because of these factors, wound repair depends on the proper coordination of several biological events. These include basic antimicrobial activities, the control and resolution of inflammation, protection against oxidative damage, the rebuilding of collagen structures, and the development of new vascular networks. Traditional Chinese Medicine (TCM) provides many active compounds. These compounds work on many targets and through different pathways. They show good potential in wound treatment. But many TCM compounds have poor solubility in water. They are also unstable, have low bioavailability, and do not pass through the skin easily. These problems limit their use in clinical settings. Nanotechnology offers new ways to solve these problems. Nanodelivery systems can improve the solubility and stability of active compounds. They can also help the compounds enter the skin and stay in the wound area. Many types of nanocarriers have been developed, such as liposomes, polymer nanoparticles, nanogels, and inorganic nanomaterials. These systems can also provide controlled release or release that responds to the wound environment. This can make the treatment more accurate. In this review, we summarize how major TCM-derived compounds support wound repair and describe the biological mechanisms behind their effects. We also discuss recent nanodelivery approaches that aim to strengthen these therapeutic actions. These combinations can improve antibacterial performance, shape the immune response, reduce reactive oxygen species, and help the skin close more quickly. We also point out several challenges, such as concerns about material safety, the need for more consistent herbal extraction methods, gaps in mechanistic understanding, and the difficulty of producing these formulations on a large scale. Taken together, these points suggest that nanodelivery approaches using TCM-derived compounds still need more careful study and steady improvement before they can be used more widely in wound care.

## 1. Introduction

Chronic wounds, such as diabetic foot ulcers and pressure ulcers, are defined as wounds in which the healing process is disrupted and fails to proceed through the normal temporal sequence of repair [1]. A key pathological feature of chronic wounds is prolonged and dysregulated inflammation, which disrupts the orderly progression of healing, leading to delayed healing and imposing a substantial healthcare burden. The refractory nature of chronic wounds is rooted in a complex pathological network characterized by several interrelated features: (i) persistent infection driven by biofilm formation [2], (ii) unresolved chronic inflammation caused by dysregulated immune responses, (iii) excessive production of reactive oxygen species (ROS) leading to oxidative stress-induced tissue damage, and (iv) insufficient angiogenesis essential for tissue regeneration [1,3]. These factors interact synergistically to form a vicious cycle, rendering conventional single-target therapeutic strategies ineffective [4].

Normal wound healing is a highly coordinated and dynamic process that unfolds in three sequential yet overlapping phases: inflammation, proliferation, and remodeling [4,5]. The initial inflammatory phase is triggered immediately after injury. It involves hemostasis and the recruitment of immune cells, such as neutrophils and macrophages, to eliminate pathogens and clear necrotic debris. This critical step creates a clean wound bed and establishes a foundation for subsequent repair. Following this, the proliferative phase commences, marked by the formation of granulation tissue. This phase is characterized by robust angiogenesis to restore blood supply, fibroblast proliferation accompanied by abundant collagen deposition to rebuild the extracellular matrix, and keratinocyte migration and proliferation to achieve re-epithelialization. The final remodeling phase is a prolonged process involving the continuous reorganization, cross-linking, and realignment of collagen fibers. This maturation of the extracellular matrix leads to a gradual increase in tensile strength and the restoration of near-normal tissue architecture [5]. Chronic wounds develop when this orderly progression is derailed.

The transition from the inflammatory to the proliferative phase is a pivotal checkpoint, and its failure is the hallmark of chronic wounds. This failure is often driven and sustained by an adverse local microenvironment. Key interfering factors that extend the inflammatory phase and hinder tissue repair include persistent microbial infection, which fuels ongoing inflammation; dysregulated immune responses leading to an overabundance of pro-inflammatory cytokines (e.g., TNF-α, IL-1β); excessive and sustained production of ROS causing oxidative damage to cells and the extracellular matrix; and elevated levels of proteases, such as matrix metalloproteinases (MMPs), which degrade newly formed granulation tissue and growth factors [3,5]. These local factors collectively create a hostile environment that prevents healing from progressing in an orderly manner. 

Traditional Chinese Medicine (TCM) has a long history in wound management, with its core strength lying in a holistic regulatory paradigm based on multi-component, multi-target, and multi-pathway regulation [6]. Accumulating studies have demonstrated that bioactive components derived from TCM—such as flavonoids, alkaloids, and saponins—can act on multiple pathological aspects of chronic wounds, including inhibiting pathogenic microorganisms, alleviating inflammation and oxidative damage, and promoting angiogenesis and cell migration. However, despite their promising therapeutic potential, these active compounds face common translational bottlenecks in clinical application. Many of them suffer from poor aqueous solubility, low chemical stability, limited bioavailability in vivo, and inadequate skin penetration [7], which severely restricts their therapeutic efficacy and broader clinical use.

**Figure 1:**
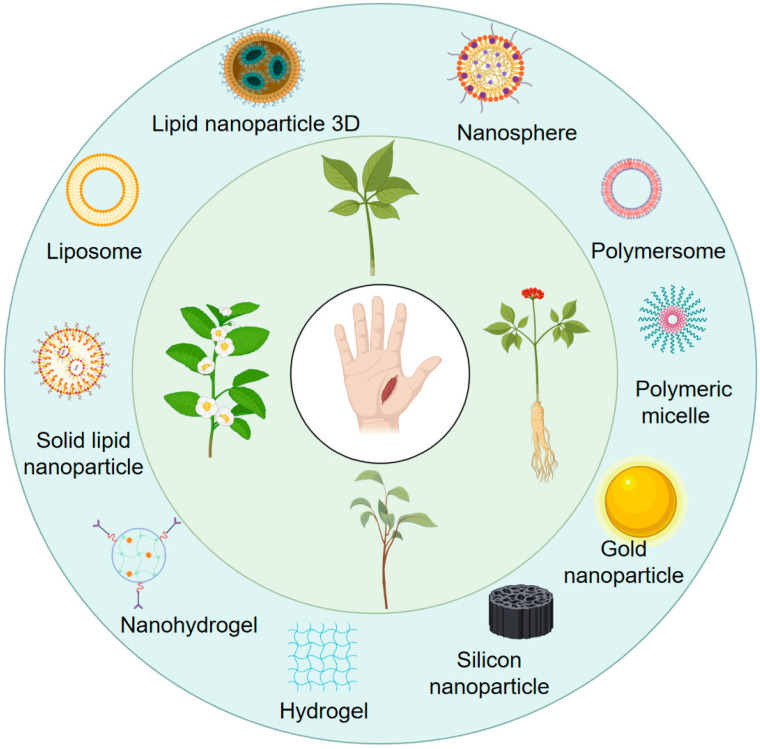
Schematic diagrams of different nanoscale drug delivery systems and nanoscaffolds.

Nanotechnology-based drug delivery systems provide a transformative approach to overcoming the limitations of TCM (Figure 1). By encapsulating or loading bioactive ingredients into nanocarriers—such as liposomes, polymeric nanoparticles, and nanogels—their solubility and stability can be significantly enhanced, while protection against premature degradation is achieved [8,9]. Moreover, nanoscale effects and surface modifications facilitate transdermal drug penetration and promote localized accumulation at wound sites. Importantly, the rational design of stimuli-responsive nanoplatforms, including pH-sensitive and enzyme-responsive systems, enables controlled or targeted drug release within the wound microenvironment, thereby improving therapeutic precision and minimizing adverse effects [10].

Several recent reviews have addressed either the pharmacological activities of TCM-derived bioactive compounds in wound healing or the engineering aspects of nanocarrier systems. However, these discussions often treat biological mechanisms and material design as parallel themes rather than as an integrated, pathology-oriented framework. In particular, explicit linkage among chemical structure features, nanocarrier design logic, and wound microenvironment-driven mechanistic regulation remains insufficiently structured. In contrast, the present review aims to provide a mechanism-centered and translationally oriented synthesis that bridges molecular structure, carrier engineering, and pathological processes in chronic wounds.

Specifically, we first summarize how representative TCM-derived bioactive compounds regulate key pathological processes in chronic wounds, including infection and biofilm persistence, dysregulated inflammation, oxidative stress imbalance, impaired cell proliferation/collagen remodeling, and insufficient angiogenesis. We then discuss how various nanocarrier platforms and smart delivery systems can be rationally designed to enhance compound stability, penetration, retention, and microenvironment-responsive release. Beyond summarizing individual studies, this review integrates mechanistic insights, structural considerations, and translational challenges—including safety evaluation, standardization, manufacturability, and clinical positioning—into a unified conceptual framework. Through this integrated perspective, we seek to provide a clearer rationale for the development of TCM-assisted nanotherapeutics for wound repair.

## 2. Mechanisms of Wound Healing Mediated by TCM-Derived Bioactive Compounds

**Figure 2:**
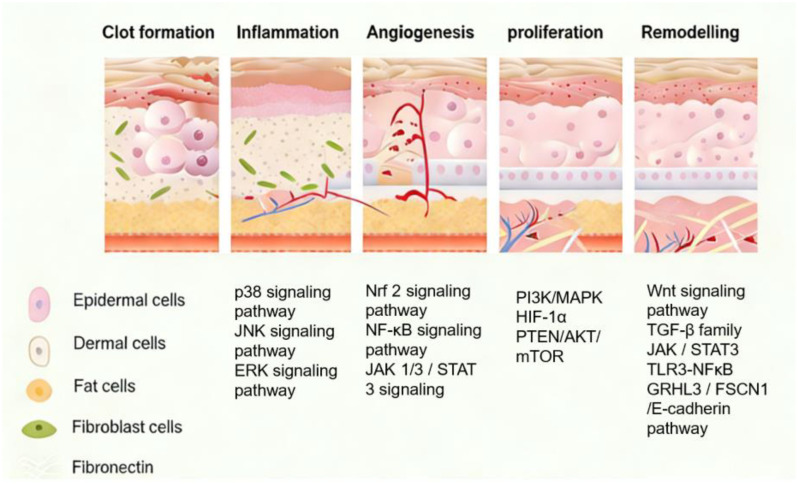
Wound healing occurs through five dynamically interconnected stages—clot formation, inflammation, angiogenesis, cell migration and proliferation, and tissue remodeling—collaboratively repairing damage and restoring tissue functionality [4,11]. (Redrawn and adapted from Shen et al. [4] under the Creative Commons Attribution-NonCommercial 4.0 International License (CC BY-NC 4.0)).

Impaired healing in chronic wounds often arises from a series of interconnected and self-sustaining pathological processes, including persistent inflammation, microbial biofilm formation, excessive oxidative stress, insufficient angiogenesis, and aberrant extracellular matrix (ECM) remodeling [2]. Within such a complex and dynamic pathological network, therapeutic strategies that target a single pathway are frequently ineffective. Notably, many bioactive compounds derived from TCM exhibit a distinctive advantage—rather than acting on an isolated target, they are capable of exerting multilevel and coordinated regulatory effects across the continuous pathological cascade of infection–inflammation–oxidative stress–tissue repair (Figure 2). In the following sections, the key pharmacological mechanisms by which these TCM-derived compounds promote wound healing are discussed in detail. The chemical structures and corresponding TCM botanical sources of the major natural compounds discussed in this review are summarized in Table 1.

### 2.1. Antibacterial and Anti-Infection Effects

Biofilm-associated infections represent a primary barrier to the healing of chronic wounds [12]. Many TCM-derived bioactive compounds not only directly inhibit bacteria but also exhibit a unique capacity to disrupt mature biofilms and reverse bacterial resistance through a variety of mechanisms.

Flavonoids, such as baicalin and quercetin, can directly compromise bacterial cell membrane integrity while also effectively suppressing bacterial quorum sensing systems, thereby interfering with biofilm formation and stability. For instance, baicalin has been shown to downregulate the expression of the *agr* quorum-sensing genes in *Staphylococcus aureus*, leading to reduced toxin production and enhanced antibiotic penetration [13]. Beyond flavonoids, other classes of TCM constituents also demonstrate the ability to interfere with bacterial communication. Emodin, an anthraquinone derivative, has been reported to significantly downregulate the expression of biofilm-associated genes *icaA* and *sarA* in *S. aureus*, thereby inhibiting the synthesis of polysaccharide intercellular adhesin (PIA) and preventing early biofilm adhesion and maturation [14]. The *icaA* gene (intercellular adhesin gene A) encodes an N-acetylglucosaminyltransferase, the key enzyme responsible for synthesizing the PIA polysaccharide backbone. The *sarA* gene (staphylococcal accessory regulator A) encodes a global regulatory protein (SarA) that acts as a positive transcriptional regulator of the ica operon, directly binding to the *icaA* promoter region to activate PIA production and biofilm formation. Phenolic acids and terpenoids, including paeonol and gallic acid, can inhibit bacterial adhesion and disrupt extracellular polysaccharide production, thus preventing initial biofilm colonization and destabilizing formed biofilm structures [15]. Alkaloids such as berberine and coptisine primarily exert membrane-targeting effects, inserting into and disrupting the bacterial phospholipid bilayer and inducing cytoplasmic leakage. Notably, berberine has also been shown to inhibit bacterial efflux pump activity, thereby acting synergistically with conventional antibiotics to overcome antimicrobial resistance [16]. Collectively, these findings illustrate the diversity of antibacterial strategies employed by TCM-derived compounds. Flavonoids like baicalin primarily target quorum sensing to disrupt biofilm communication, whereas alkaloids such as berberine act directly on membrane integrity and efflux pumps. Notably, while both approaches are effective, membrane-targeting agents may exhibit broader-spectrum activity but raise cytotoxicity concerns at higher concentrations. In contrast, compounds that modulate gene expression (e.g., emodin downregulating *icaA* and *sarA*) offer a more targeted approach to biofilm inhibition but require longer exposure times. However, a common limitation across these studies is the predominant focus on *S. aureus*, leaving efficacy against polymicrobial biofilms—more clinically relevant in chronic wounds—largely unexplored. Future research should prioritize combination therapies that leverage the synergistic potential of these diverse mechanisms to overcome biofilm heterogeneity and combat antimicrobial resistance.

### 2.2. Modulation of Inflammation and Immune Responses

The central pathological impediment to chronic wound healing lies in dysregulated inflammation, characterized by the persistent dominance of a pro-inflammatory state and the failure to transition in an orderly manner toward the reparative phase. Under such conditions, sustained activation and accumulation of M1-polarized macrophages lead to excessive secretion of pro-inflammatory mediators, including tumor necrosis factor-α (TNF-α) and interleukin-1β (IL-1β). These mediators not only directly damage local tissues but also impair key reparative processes such as fibroblast proliferation, collagen synthesis, and angiogenesis [17,18,19]. Accordingly, promoting the phenotypic transition of macrophages from the pro-inflammatory M1 phenotype to the anti-inflammatory and pro-repair M2 phenotype has emerged as a critical regulatory node for restoring inflammatory balance and re-initiating the wound-healing program in chronic wounds [20]. M2 macrophages not only suppress excessive inflammation but also directly facilitate angiogenesis, extracellular matrix remodeling, and re-epithelialization through the release of cytokines such as interleukin-10 (IL-10) and transforming growth factor-β (TGF-β) [21].

A growing number of TCM has demonstrated the capacity to precisely regulate this immune transition, with mechanisms involving modulation of multiple signaling pathways. Numerous studies indicate that compounds such as ginsenoside Rg1 and astragaloside IV promote macrophage polarization toward the M2 phenotype by activating signaling pathways including AMPK and STAT6, thereby reducing pro-inflammatory cytokine production while enhancing the release of reparative factors [22]. For example, astragaloside IV has been shown to significantly upregulate the expression of the M2 marker arginase-1 (Arg-1) and increase IL-10 secretion through activation of the STAT6 pathway, effectively improving the inflammatory microenvironment in diabetic wound models [23]. In addition, compounds such as paeonol and celastrol have also been reported to promote M2 polarization via modulation of the PPAR-γ pathway [24,25].

On the other hand, certain TCM constituents mitigate chronic inflammation by suppressing key upstream pro-inflammatory signaling networks, thereby restraining the amplification of the so-called “cytokine storm” and creating a permissive environment for macrophage phenotypic switching. Curcumin and resveratrol are well-recognized inhibitors of the NF-κB and NLRP3 inflammasome pathways and can effectively block the cascade amplification of inflammatory signaling [26]. Studies have shown that curcumin analogs directly inhibit the assembly and activation of the NLRP3 inflammasome, reducing the maturation and release of IL-1β and alleviating the sustained pro-inflammatory state of macrophages [27]. Panax notoginseng saponins have also been reported to downregulate M1-associated gene expression by blocking the nuclear translocation of NF-κB. Notably, these compounds may exert enhanced effects when used in combination. For instance, co-administration of coptisine and berberine has been shown to more effectively drive macrophage polarization toward a reparative phenotype through multi-target inhibition of inflammatory pathways [28].

The immunomodulatory effects of TCM compounds reveal two predominant yet complementary strategies. One strategy, exemplified by ginsenoside Rg1 and astragaloside IV, actively promotes macrophage polarization toward the pro-repair M2 phenotype via pathways such as STAT6. In contrast, compounds like curcumin and resveratrol adopt a “passive” approach by suppressing pro-inflammatory signaling (e.g., NF-κB and NLRP3 inflammasome), thereby facilitating phenotypic switching. Of note, while M2 polarization is desirable, its optimal therapeutic window remains unclear; excessive or premature M2 skewing might impair pathogen clearance. However, most current evidence derives from single-compound studies. In contrast, combination therapies, such as coptisine and berberine co-administration [28], suggest superior efficacy through multi-target inhibition, indicating that synergistic formulations may better restore immune homeostasis in complex chronic wound environments.

### 2.3. Antioxidant Effects and ROS Scavenging

During normal wound healing, ROS function as essential signaling molecules that coordinate and drive the orderly progression of tissue repair. Beyond serving as secondary messengers within immune and non-immune cells, ROS participate in the regulation of cell proliferation, migration, and differentiation, and play indispensable roles in directing lymphocyte recruitment to the injury site as well as modulating local angiogenesis and blood perfusion. In contrast, in chronic wounds such as diabetic foot ulcers, venous or arterial leg ulcers, and pressure ulcers, an imbalance between ROS generation and clearance leads to their persistent and excessive accumulation within the wound microenvironment. Under these conditions, ROS shift from being physiological regulators of repair to pathological mediators that induce oxidative stress, damage cellular structures, and ultimately impede the healing process [29]. Consequently, precise modulation of ROS levels and restoration of redox homeostasis have emerged as critical strategies for improving the wound-healing microenvironment in chronic wounds.

Against this background, the rich repertoire of naturally occurring antioxidant constituents in TCM offers multilevel and multitarget opportunities for achieving precise redox regulation. On the one hand, many TCM-derived bioactive compounds, particularly flavonoids (such as rutin and luteolin) and phenolic acids, possess phenolic hydroxyl groups within their molecular structures that enable them to directly donate electrons, thereby efficiently neutralizing reactive oxygen species, including superoxide anions (O_2_^−^) and hydroxyl radicals (·OH) through direct scavenging mechanisms [30]. On the other hand, certain constituents, such as tanshinone IIA and apigenin, exert antioxidant effects primarily by activating key intracellular antioxidant signaling pathways, most notably the nuclear factor erythroid 2-related factor 2 (Nrf2) pathway. Activation of Nrf2 subsequently upregulates the transcription and expression of multiple endogenous antioxidant enzymes, including superoxide dismutase (SOD) and glutathione peroxidase (GPx), thereby systemically enhancing the capacity of tissue cells to resist oxidative injury [31].

In addition, some TCM components reduce excessive ROS generation through indirect mechanisms, such as modulation of mitochondrial function and inhibition of inflammation-associated enzymes, including NADPH oxidase. These diverse antioxidant actions function synergistically to form a multilayered defensive network. TCM-derived antioxidants operate through distinct yet complementary mechanisms. Direct radical scavengers (e.g., flavonoids, phenolic acids) provide rapid, direct ROS neutralization. In contrast, compounds like tanshinone IIA activate the Nrf2 pathway to induce sustained antioxidant enzyme responses. Of note, these strategies are synergistic: direct scavengers offer immediate defense, while Nrf2 activators enhance long-term cellular resilience. However, a key limitation is the over-reliance on cell-free or simple cell-based assays. In contrast, studies examining ROS modulation within the complex chronic wound microenvironment—where precise tuning, not complete elimination, of ROS is required—remain scarce. Future research should prioritize achieving redox balance over maximal ROS scavenging.

### 2.4. Promotion of Cell Proliferation and Collagen Synthesis

During the proliferative phase of wound healing, the timely activation and coordinated function of fibroblasts and keratinocytes constitute the cellular basis for tissue regeneration. Fibroblasts are responsible for the synthesis and secretion of collagen and other ECM components, thereby establishing the structural framework of granulation tissue, whereas keratinocytes migrate and proliferate to achieve re-epithelialization and complete wound coverage [32]. Accumulating evidence indicates that multiple TCM-derived bioactive compounds can effectively facilitate these reparative processes by modulating distinct molecular signaling networks. For instance, Panax notoginseng saponins and icariin have been shown to directly promote the proliferation and migration of fibroblasts and keratinocytes through activation of key signaling pathways such as PI3K/Akt and Wnt/β-catenin [33]. Activation of the PI3K/Akt pathway suppresses apoptosis and enhances cellular metabolic activity, whereas stimulation of the Wnt/β-catenin pathway upregulates cell cycle-related proteins, including cyclin D1, thereby driving cell cycle progression. In addition, compounds such as ginsenoside Rb1 have been reported to enhance the expression of adhesion molecules, including integrin β1, strengthening cell adhesion to ECM components such as fibronectin and laminin, and providing the anchorage and traction forces required for effective cell migration [34].

At the more complex level of ECM synthesis, assembly, and remodeling, TCM exhibits multilayered regulatory capabilities. Asiaticoside can directly stimulate the production of TGF-β1, thereby upregulating the transcription and synthesis of type I and type III collagen. Concurrently, it downregulates the activity of matrix-degrading enzymes such as MMP-1 and MMP-3 while upregulating their endogenous inhibitor TIMP-1, fine-tuning the MMP/TIMP balance to prevent excessive collagen degradation and promote granulation tissue maturation and reinforcement [35]. Similarly, astragaloside IV has been identified as an effective activator of the TGF-β/Smad signaling pathway. By promoting the phosphorylation and nuclear translocation of Smad2/3, astragaloside IV directly initiates the transcription of multiple ECM-related genes, including collagen I, collagen III, and fibronectin, thereby actively facilitating collagen synthesis, secretion, and orderly deposition [36]. The pro-regenerative effects of TCM compounds converge on key cellular processes through distinct molecular routes. Some, like Panax notoginseng saponins, primarily drive cell cycle progression via the PI3K/Akt pathway. In contrast, others, such as asiaticoside and astragaloside IV, modulate extracellular matrix dynamics via the TGF-β/Smad axis and MMP/TIMP balance. Of note, while collagen synthesis is beneficial, matrix quality is equally critical. Asiaticoside’s ability to fine-tune MMP/TIMP ratios offers a regulatory advantage over compounds that merely stimulate collagen production. However, long-term studies assessing effects on pathological scarring are lacking. Promoting proliferation without ensuring proper matrix remodeling risks functional impairment of healed tissue [37].

### 2.5. Promotion of Angiogenesis

Angiogenesis represents a central determinant of functional wound repair, as an adequate blood supply is essential for sustaining cell proliferation, nutrient delivery, and extracellular matrix remodeling. A growing body of evidence indicates that numerous TCM-derived bioactive compounds act as important modulators of angiogenesis through precise regulation of multiple signaling pathways [38,39].

On the one hand, many compounds directly drive neovascularization by upregulating key angiogenic growth factors and their receptors. For example, salidroside and ferulic acid have been shown to significantly increase the expression of vascular endothelial growth factor (VEGF), basic fibroblast growth factor (bFGF), and their corresponding receptors in vascular endothelial cells, thereby directly stimulating endothelial cell function [38]. Similarly, astragaloside IV promotes angiogenesis by upregulating VEGF expression via activation of the PI3K/Akt signaling pathway [40], while icariin exerts pro-angiogenic effects through activation of the Src/PI3K/Akt axis [41].

On the other hand, in response to the hypoxic microenvironment characteristic of chronic wounds, certain TCM constituents enhance angiogenic capacity by stabilizing core hypoxia-responsive transcription factors. Xanthotoxin has been reported to promote sustained transcription of downstream angiogenic genes by stabilizing hypoxia-inducible factor-1α (HIF-1α) [39]. Likewise, celastrol has been demonstrated to robustly stimulate angiogenesis and accelerate wound healing through activation of the HIF-1α/VEGF signaling axis [42].

Beyond these mechanisms, the pro-angiogenic actions of TCM-derived compounds extend to coordinated regulation across diverse signaling networks. For instance, notoginsenoside R1 promotes vascularization through activation of the Wnt/β-catenin pathway [43], whereas ginsenoside Rg1 enhances angiogenesis under ischemic conditions by activating the Nrf2/HO-1 pathway [44]. TCM compounds promote angiogenesis through interconnected signaling pathways with distinct regulatory advantages. Many, like salidroside and astragaloside IV, directly upregulate VEGF, providing potent endothelial stimuli. In contrast, compounds such as xanthotoxin stabilize HIF-1α, coupling angiogenesis with metabolic adaptation. Of note, multi-functional compounds like berberine and luteolin combine pro-angiogenic with anti-inflammatory and antioxidant effects. In contrast, this integrated profile may be particularly advantageous for chronic wounds, where angiogenesis is impaired by a hostile microenvironment rather than a single deficit. However, the lack of standardized metrics for comparing pro-angiogenic potency hindered candidate prioritization for clinical translation [45]. Furthermore, flavonoids such as luteolin have also been demonstrated to amelio-rate impaired angiogenesis in diabetic wounds via regulating endothelial cell prolifer-ation and migration [46].

A comparative overview of the primary biological mechanisms, advantages, and limitations of representative TCM-derived compounds in wound healing is provided in Table 2.

## 3. Nanocarrier Systems for the Delivery of TCM Active Constituents

### 3.1. Lipid-Based Nanocarriers

Among the various nanocarrier platforms, lipid-based nanocarriers exhibit distinct advantages owing to their structural similarity to biological membranes. Liposomes, composed of phospholipid bilayers, represent the most classical and extensively studied model within this category [47]. Their architecture mimics that of natural cell membranes, featuring an aqueous core capable of encapsulating hydrophilic drugs, while the lipid bilayer itself accommodates lipophilic molecules, thereby enabling the simultaneous loading of both hydrophilic and hydrophobic agents [48]. This unique structural organization endows liposomes with excellent biocompatibility and biodegradability. Moreover, their surface can be readily functionalized through chemical modifications—such as polyethylene glycol (PEG) conjugation to prolong systemic circulation or the grafting of targeting peptides or antibodies to enhance tissue specificity—thereby markedly improving targeted drug delivery efficiency [49]. In the context of wound therapy, liposomes not only serve as efficient transdermal delivery vehicles [50], but their phospholipid components themselves also contribute positively to membrane repair and the maintenance of skin barrier function.

#### 3.1.1. Solid Lipid Nanoparticles

To overcome the limitations of conventional liposomes, particularly with respect to encapsulation efficiency and long-term storage stability, solid lipid nanoparticles (SLNs) were subsequently developed. These nanoparticles are constructed using lipids that remain solid at physiological temperature such as glyceryl monostearate to be the core matrix, thereby forming a more structurally stable solid lipid framework [51]. This solid-state matrix effectively restricts premature drug leakage during storage and in vivo delivery, resulting in a more controllable release profile. Consequently, SLNs are especially suitable for therapeutic applications requiring sustained and prolonged drug release [52]. However, the highly ordered crystalline structure of solid lipids also imposes inherent limitations. The available space for drug accommodation is relatively restricted, and during particle formation, drug molecules may be partially expelled from the tightly packed crystal lattice. As a result, SLNs may exhibit limited drug-loading capacity, as well as batch-to-batch variability in encapsulation efficiency [53].

#### 3.1.2. Nanostructured Lipid Carriers

To address the intrinsic limitations of solid lipid nanoparticles, nanostructured lipid carriers (NLCs) were subsequently developed. The fundamental design strategy of NLCs involves incorporating a certain proportion of liquid lipids (e.g., medium-chain triglycerides such as caprylic/capric triglyceride) into the solid lipid core. The presence of liquid lipids disrupts the highly ordered crystalline arrangement typically formed by solid lipids, resulting in a less regular, partially amorphous lipid matrix with numerous imperfections and voids [54]. This deliberately “imperfect” internal structure provides additional space for drug accommodation. Consequently, NLCs retain the favorable characteristics of SLNs—such as good physical stability and controlled drug release—while markedly improving the loading capacity and encapsulation efficiency for hydrophobic compounds, as well as reducing drug expulsion during storage [55].

Owing to their lipid-based composition, NLCs exhibit strong affinity with the lipid components of the stratum corneum. This feature enables them to function not only as efficient nanocarriers that facilitate the transdermal delivery of poorly water-soluble drugs [56], but also as auxiliary wound dressings after topical application. Upon administration to the wound surface, NLCs can contribute to the formation of a protective lipid film, which helps maintain a moist wound environment, reduce transepidermal water loss, and create favorable conditions for cell migration and proliferation [57].

The therapeutic potential of NLC-based systems has been substantiated by numerous application studies. For instance, ginsenoside Rg1 has been encapsulated into lipid-based nanocarriers and subsequently incorporated into a sodium carboxymethyl cellulose gel to fabricate a composite wound dressing [58]. This delivery system significantly enhanced transdermal drug penetration and increased drug retention across different skin layers. In diabetic rat wound models, the sustained-release formulation promoted collagen deposition, angiogenesis, and re-epithelialization by upregulating key mediators such as VEGF, resulting in markedly superior healing outcomes compared with gels containing free Rg1 [58].

Similarly, studies on ginsenoside Rg3 have demonstrated that formulation into solid lipid nanoparticle-based gels confers enhanced therapeutic efficacy. Benefiting from improved stability, sustained drug release, and superior skin delivery efficiency, the Rg3-loaded system exerted pronounced anti-inflammatory effects—characterized by reduced TNF-α levels and elevated IL-10 expression—while simultaneously promoting tissue repair in murine wound models. These combined effects effectively improved the wound microenvironment and accelerated granulation tissue formation [59]. Consistent enhancements in wound healing have also been reported for other poorly soluble TCM-derived active compounds, including resveratrol and curcumin, following their incorporation into NLC-based delivery platforms [60].

Lipid-based nanocarriers are widely explored for wound therapy because they can enhance topical delivery and local retention of poorly water-soluble bioactives and support multifunctional designs. Nevertheless, their clinical translation is frequently limited by formulation stability and reproducible product quality. Liposomal systems may undergo leakage, aggregation/fusion, and lipid oxidation during storage and handling, requiring systematic stability characterization and well-defined critical quality attributes for development and release testing [61]. For SLNs/NLCs, solid–liquid lipid matrix organization and polymorphic transitions can affect encapsulation and release behavior over time, which is particularly relevant for wound products that must remain stable under practical storage conditions and during repeated dressing changes. In addition, the protein- and enzyme-rich wound milieu may alter lipid nanostructures and reduce the predictability of conventional in vitro release/penetration assays for in situ performance [62]. Therefore, future studies should prioritize wound-relevant stability and performance benchmarking, standardized quality attributes, and clinically meaningful comparators to facilitate translation of lipid-based platforms into robust wound-care formulations.

### 3.2. Polymer-Based Nanocarriers

Polymer-based nanocarriers represent another highly promising class of drug delivery platforms. Owing to their versatile material choices and flexible design strategies, these systems can be tailored to address the complex and stage-specific requirements of wound healing. In the following sections, several representative polymeric materials and their functional design strategies are discussed [63].

#### 3.2.1. Poly(Lactic-co-glycolic Acid)

Poly(lactic-co-glycolic acid) (PLGA) is an FDA-approved biodegradable synthetic polymer that has attracted considerable attention in wound therapy, primarily due to its outstanding capacity for controlled drug release [60]. A key advantage of PLGA lies in the precise tunability of its degradation rate and drug release kinetics by adjusting the molar ratio of its monomeric components, lactic acid and glycolic acid. This feature enables programmed drug release over a time frame ranging from several weeks to months, making PLGA-based systems particularly suitable for the long-term management of chronic wounds [64]. Moreover, PLGA undergoes complete degradation in vivo into carbon dioxide and water, conferring excellent biocompatibility and a well-established safety profile [65]. Beyond serving as a vehicle for small-molecule drugs, PLGA nanoparticles can also function as protective carriers for labile bioactive macromolecules, such as epidermal growth factor (EGF) and bFGF. By shielding these growth factors from premature degradation in the protease-rich wound environment, PLGA-based delivery systems enable their sustained and stable bioactivity, thereby enhancing their pro-regenerative effects [65].

#### 3.2.2. Chitosan

Chitosan, a naturally derived cationic polysaccharide, is widely recognized not only for its excellent biocompatibility and biodegradability but also for its intrinsic bioactivities related to hemostasis and wound healing [66]. Owing to its positive surface charge, chitosan can readily interact with negatively charged bacterial cell membranes and the stratum corneum via electrostatic attraction. This property contributes to enhanced antibacterial effects and, at the same time, improves transdermal drug permeation and local retention at the wound site [66]. Recent studies have further revealed that the degradation product of chitosan, N-acetylglucosamine, serves as a key precursor for hyaluronic acid biosynthesis. Through this pathway, it can directly stimulate fibroblast activity and promote extracellular matrix production, thereby actively participating in the tissue repair process [67]. Chitosan nanoparticles prepared via ionic crosslinking with sodium tripolyphosphate (STPP) represent a particularly versatile delivery system. These nanoparticles are capable of efficiently encapsulating protein-based therapeutics and can undergo responsive swelling in the presence of wound exudates, enabling stimulus-responsive drug release. Such properties render chitosan-based nanocarriers highly promising as multifunctional wound dressing matrices that integrate drug delivery with inherent pro-healing functions [68].

#### 3.2.3. Hyaluronic Acid

Hyaluronic acid (HA) is a major component of the extracellular matrix and, as a naturally derived polymer, exhibits distinct advantages in the design of nanocarrier systems for wound-healing applications [69]. HA can specifically bind to CD44 receptors expressed on the surface of key wound-healing-associated cells, such as fibroblasts and macrophages, thereby endowing HA-based nanoparticles with intrinsic targeting capability. In addition, HA actively participates in the regulation of inflammatory responses and the maintenance of tissue hydration, both of which are critical for effective wound repair [69]. Notably, the biological functions of HA are highly dependent on its molecular weight. High-molecular-weight HA exerts anti-inflammatory and immunomodulatory effects during the early inflammatory phase, whereas low-molecular-weight HA fragments have been shown to promote angiogenesis [70]. By exploiting these properties, HA-based nanocarriers not only enable the targeted delivery of therapeutic agents to CD44-overexpressing activated repair cells but also function as bioactive signaling molecules that directly modulate the wound-healing process. For example, hydrophobically modified HA can self-assemble into nanomicelles capable of encapsulating poorly soluble anti-inflammatory drugs, thereby achieving a synergistic “carrier–drug” dual therapeutic effect [71].

#### 3.2.4. Functional Block Copolymers

Beyond the conventional polymeric materials discussed above, functional block copolymers engineered through rational molecular design have emerged as a distinct and innovative class of nanocarriers. For example, Li et al. reported a block copolymer, DA95B5, that can self-assemble into nanoparticles featuring a non-adhesive dextran shell and a cationic core [72]. These nanoparticles are capable of penetrating and associating with biofilms formed by various multidrug-resistant bacteria. Importantly, their primary mode of action is not direct bactericidal activity. Instead, the dextran shell enhances the solubility of bacteria–nanoparticle complexes, thereby facilitating the gradual detachment and diffusion of bacterial cells from the biofilm matrix. Through this mechanism, the structural integrity of mature biofilms is progressively disrupted, leading to effective biofilm dispersion rather than outright bacterial killing. This “biofilm-dispersal” strategy offers a fundamentally different approach to addressing persistent biofilm-associated infections in chronic wounds. By avoiding strong bactericidal pressure, it may reduce the selective drive for the development of bacterial resistance [73]. When combined with conventional antimicrobial agents, such functional polymers can first dismantle the biofilm architecture and subsequently enhance the clearance of released planktonic bacteria, achieving a synergistic therapeutic effect.

Polymer-based nanocarriers offer highly tunable chemistry and release profiles for wound therapy, enabling the integration of antimicrobial, immunomodulatory, and pro-regenerative functions into a single platform. However, translation is frequently constrained by reproducibility and quality control, particularly when using natural polymers whose molecular weight distribution, degree of substitution/deacetylation, and impurity profiles can vary across sources and processing routes, thereby affecting particle formation, drug loading, and biological performance [6,74]. For synthetic polyesters such as PLGA, degradation kinetics are formulation- and environment-dependent; the generation of acidic byproducts and local microenvironmental acidification may influence payload stability and host response, which is especially relevant in inflamed and protease-rich wound beds where in situ degradation and release may deviate from simplified in vitro conditions [62,75]. In addition, increasing structural complexity can improve performance in proof-of-concept studies but often raises barriers for scale-up manufacturing, sterilization compatibility, and definition of critical quality attributes needed for robust product release. Therefore, future development should prioritize wound-relevant testing conditions, clearly defined material specifications and CQAs, and scalable fabrication routes that balance functional sophistication with manufacturability.

### 3.3. Nanogels and Hydrogels

Gel-based systems play an indispensable role in wound care due to their high water content and excellent biocompatibility. Depending on their structural scale and primary function, they can be broadly classified into nanogels, which mainly focus on intracellular drug delivery, and macroscopic hydrogels, which serve as multifunctional wound dressing matrices. These two systems can be applied independently or integrated to construct more efficient multilevel delivery platforms [76].

#### 3.3.1. Nanogels

Nanogels are three-dimensional crosslinked polymeric networks with nanoscale dimensions (typically 10–200 nm), formed from hydrophilic or amphiphilic polymers. Their key advantage lies in the combination of the size-related benefits of nanoparticles with the high drug-loading capacity and stimulus responsiveness characteristic of gel systems [77]. The nanoscale size of nanogels facilitates their accumulation at wound sites via enhanced permeability and retention-like effects and enables efficient cellular uptake by reparative cells, thereby allowing precise intracellular drug delivery [78]. By incorporating environmentally sensitive crosslinkers, smart nanogels can be engineered to undergo stimulus-triggered drug release in response to specific cues, such as pH changes, redox conditions, or enzymatic activity. This property makes nanogels particularly well suited for the targeted modulation of intracellular pathways involved in wound healing [79].

#### 3.3.2. Hydrogels

Macroscopic hydrogels represent another distinctive class of integrated delivery and dressing platforms, characterized by their hydrophilic three-dimensional network structures [80]. Such networks can absorb and retain large amounts of water, thereby creating an optimal moist wound-healing environment. This not only prevents tissue dehydration and necrosis but also facilitates cell migration and re-epithelialization [80]. In parallel, the open and porous architecture of hydrogels ensures adequate gas exchange and excellent biocompatibility. Their physicochemical properties, including stiffness and porosity, can be flexibly tuned by selecting different polymeric components—such as gelatin, alginate, or cellulose derivatives—and adjusting the crosslinking density [80]. Consequently, hydrogels function not only as physical barriers protecting the wound bed but also as effective local drug reservoirs.

One of the most advanced application strategies involves embedding pre-fabricated drug-loaded nanoparticles (e.g., liposomes or polymeric nanoparticles) within hydrogel matrices to construct hierarchical delivery systems [81]. For instance, liposomes encapsulating shikonin, which possesses both anti-inflammatory and pro-angiogenic activities, have been homogeneously dispersed in a thermosensitive chitosan/β-glycerophosphate hydrogel precursor solution [82]. Upon contact with the wound at body temperature, the system rapidly undergoes sol–gel transition to form a protective dressing. Beyond providing a favorable moist environment and mechanical protection, the hydrogel network acts as a diffusion-regulating barrier that effectively retards the release of the embedded liposomes, enabling sustained and controlled drug delivery. Animal studies demonstrated that this nanoparticle–hydrogel composite dressing maintained therapeutically relevant drug concentrations at the wound site over extended periods and significantly accelerated full-thickness skin wound healing through the combined effects of moisture retention, inflammation suppression, and angiogenesis promotion, outperforming liposomes or blank hydrogels used alone [82].

Another advanced strategy is the development of cascade-responsive, self-activating antibacterial hydrogels. These systems are capable of sensing infection-associated microenvironmental changes, such as local acidification or the presence of specific enzymes, to trigger the on-demand release of antimicrobial agents and activate nanozyme-catalyzed reactions for efficient elimination of bacteria and biofilms. Subsequently, the hydrogel adaptively transitions into a reparative mode, continuously alleviating oxidative stress and promoting angiogenesis to support tissue regeneration [83].

Hydrogel- and gel-based systems are highly attractive for wound management because they provide a moist microenvironment, barrier protection, and exudate handling while serving as local depots for sustained or triggered delivery. However, the multifunctionality that makes hydrogels clinically appealing also complicates mechanistic interpretation and translational evaluation. Specifically, improvements in wound closure are often driven by the dressing matrix itself, which can obscure the incremental contribution of the embedded nanoparticles or TCM-derived payloads unless rigorous comparator controls are included [84,85]. Moreover, in vitro release profiles and cell-based assays may poorly reflect in situ performance because wound beds exhibit pronounced temporal and spatial heterogeneity in exudate volume, protease burden, bacterial biofilms, and dressing-change frequency—factors that can substantially alter gel degradation, drug diffusion, and local exposure [85]. From a clinical translation perspective, practical considerations such as sterilization compatibility, storage stability, conformability/adhesion, painless removal, and consistent performance across wound types are frequently underreported despite being critical to real-world adoption. Therefore, future studies should incorporate wound-relevant testing conditions, standardized outcome measures beyond closure rate, and usability-oriented evaluation to better bridge proof-of-concept materials to deployable wound-care products [86].

### 3.4. Inorganic Nanomaterials

Nanotechnology-based systems derived from inorganic materials not only serve as drug delivery vehicles but can also directly participate in and even dominate therapeutic processes through their intrinsic physicochemical properties, such as catalytic activity, ion release, and photothermal conversion. This unique feature enables the development of multifunctional platforms that integrate both carrier and therapeutic functions, offering powerful solutions for advanced wound management [87].

#### 3.4.1. Mesoporous Silica Nanocarriers

Mesoporous silica nanoparticles (MSNs) have emerged as an ideal drug delivery platform owing to their distinctive structural characteristics. They possess highly ordered nanoscale pore channels, exceptionally large specific surface areas, and surfaces that are readily amenable to chemical functionalization. These mesoporous architectures enable efficient loading of a wide range of therapeutic cargos, including small-molecule drugs such as flavonoids and alkaloids, as well as certain biomacromolecules [88]. More importantly, surface functionalization strategies—such as grafting amino or carboxyl groups, or installing stimulus-responsive “molecular gates”—can endow MSNs with intelligent responsiveness. Such engineered MSNs are capable of sensing wound-specific microenvironmental cues, including pH fluctuations, enzymatic activity, or elevated ROS levels, thereby achieving site-specific and on-demand drug release at the lesion site [89]. For example, quercetin, a representative flavonoid compound, has been successfully loaded into MSNs to exploit their porous structure for sustained drug release. In diabetic wound models, this MSN-based delivery system exhibited pronounced antioxidant and anti-inflammatory effects, while simultaneously promoting angiogenesis and re-epithelialization. These combined actions effectively accelerated the wound-healing process, highlighting the therapeutic potential of MSNs in managing chronic and metabolically impaired wounds [89,90,91].

#### 3.4.2. Antibacterial Metal Nanoparticles

Metal nanoparticles such as silver, zinc, and copper, as well as their oxides, play a dual role in wound treatment: they function not only as drug delivery carriers but also as effective therapeutic agents themselves. Their antibacterial activity is achieved through multiple synergistic mechanisms. On the one hand, the slow release of metal ions such as silver ions (Ag^+^) and zinc ions (Zn^2+^) can disrupt microbial cell membranes and interfere with intracellular enzyme activity and DNA replication. On the other hand, under light exposure or in the presence of certain microbial metabolites, these nanoparticles can catalyze the generation of reactive oxygen species, causing oxidative damage to bacteria. In addition, the physical interactions of the nanoparticles themselves can directly disrupt the structure of bacterial biofilms [92,93].

Notably, the released metal ions also exert important pro-healing effects. Zinc ions are essential cofactors for a variety of metalloenzymes involved in cell proliferation and migration and can directly promote epithelial cell migration and collagen synthesis, while copper ions participate in signaling pathways that regulate angiogenesis [94]. For example, gallic acid has been combined with zinc oxide nanoparticles to form a composite system, which not only exhibits synergistic antibacterial activity but also significantly accelerates re-epithelialization in diabetic mouse wound models through the sustained release of zinc ions [95].

#### 3.4.3. Catalytic Nanozymes

A class of inorganic nanomaterials represented by cerium oxide (CeO_2_) and magnetite (Fe_3_O_4_) exhibits a unique “enzyme-like” activity. Depending on changes in their surface chemical states, these materials can reversibly mimic the functions of endogenous SOD and catalase (CAT), continuously catalyzing the conversion of harmful O_2_^−^ and hydrogen peroxide (H_2_O_2_) at the wound site into harmless substances. This catalytic capability is relatively insensitive to fluctuations in environmental pH, providing a stable tool for the sustained management of oxidative stress in chronic wounds [96].

Studies have shown that incorporating CeO_2_ nanozymes into hydrogels to form wound dressings can effectively protect fibroblasts at the wound site from hydrogen peroxide-induced cell death, while simultaneously directing macrophages to polarize toward the pro-repair M2 phenotype, thereby achieving both antioxidant and anti-inflammatory effects in a single intervention [97]. More recent research strategies have further expanded their applications; for example, nanozymes with glucose oxidase-like activity have been integrated with insulin delivery systems, offering a novel solution for managing the hyperglycemic microenvironment characteristic of diabetic wounds [98].

#### 3.4.4. Externally Responsive Formulations

A class of inorganic materials represented by gold nanorods, upconversion nanoparticles, and superparamagnetic iron oxide nanoparticles is characterized by its ability to efficiently absorb external energy sources—such as near-infrared (NIR) light, ultrasound, or alternating magnetic fields—and convert them into trigger signals for controlled drug release. This precise manipulation by external physical fields introduces new possibilities for achieving spatiotemporally controlled therapy: clinicians can remotely activate the system at specific time points according to the wound condition, targeting refractory lesions such as deep-seated infection foci, thereby maximizing therapeutic efficacy while minimizing damage to surrounding healthy tissues. At the same time, the local heat generated during the energy conversion process (photothermal or magnetothermal effects) constitutes a form of physical therapy in itself, capable of directly damaging bacteria and disrupting biofilm structures, and acting synergistically with the antibacterial or anti-inflammatory agents released from the nanocarriers [99,100]. Studies have demonstrated that NIR irradiation of antibiotic-loaded Au@mesoporous silica core–shell nanoparticles can simultaneously achieve photothermal bacterial killing and heat-triggered drug release, showing pronounced penetration and eradication effects against dense, mature bacterial biofilms [101]. Such systems offer a highly promising strategy for the treatment of deep, complex wound infections that are difficult to manage using conventional approaches.

Inorganic materials offer strong antibacterial and multifunctional effects that are attractive for infected and chronic wounds; however, translation requires a more cautious, safety-centered evaluation. A key challenge is the potential trade-off between antimicrobial efficacy and host–cell compatibility: metal ion release and redox activity that contribute to bacterial killing may also induce oxidative stress and reduce viability of skin and immune cells under certain conditions, highlighting the need to define a clinically relevant therapeutic window [102]. Moreover, many studies emphasize short-term outcomes, while longer-term biodistribution, degradation/clearance behavior, and subchronic/chronic toxicity remain insufficiently characterized for numerous inorganic systems, which is a major barrier for clinical development and regulatory acceptance [103,104]. In addition, the biological identity and performance of inorganic nanoparticles can be altered by wound microenvironment factors that influence surface chemistry and reactivity, potentially reducing the predictability of simplified in vitro assays [6,104]. Therefore, future work should incorporate standardized safety assessment and more explicit consideration of manufacturability and quality control to support clinically deployable inorganic nano-enabled wound therapies. The physicochemical properties, functional characteristics, and representative applications of various nanocarrier systems for TCM compound delivery are compared in Table 3.

### 3.5. Stimuli-Responsive and Smart Delivery Systems

**Figure 3:**
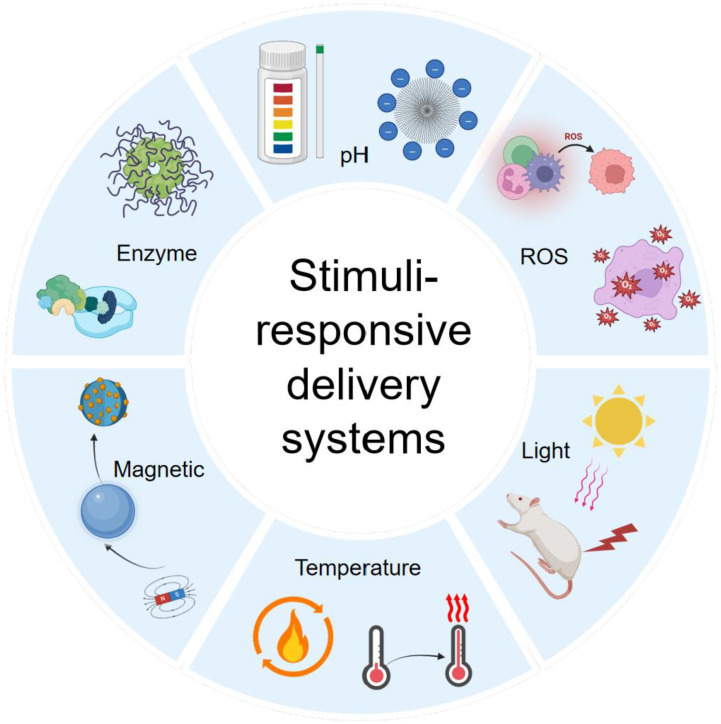
Stimuli-responsive and smart delivery systems.

The core challenge of chronic wounds lies in their establishment of a unique and persistent pathological microenvironment. This microenvironment is not chaotic; rather, it constitutes a “pathological yet relatively stable” new steady state formed by multiple dysregulated biochemical signals. For example, persistent bacterial infection and ischemia result in sustained local acidity; excessive inflammation and cell death lead to abnormally high concentrations of specific proteases, such as matrix metalloproteinases (MMPs); dysfunction of repair-cell mitochondria combined with immune cell respiratory bursts causes elevated levels of ROS; and glucose concentrations are significantly higher in diabetic wounds than normal tissues [78,105]. These signals are not only outcomes and markers of the pathological state, but also serve as temporally and spatially specific cues that persist throughout the chronic phase and are localized to the wound site, providing natural and distinguishable physiological triggers for the design of targeted smart drug delivery systems (Figure 3) [106].

Accordingly, the design concept of smart delivery systems has shifted from traditional “passive release” to “active response.” At its core, the system loads drugs into carriers capable of sensing these specific pathological signals. In normal tissue or during the benign stages of wound healing, the system remains silent, releasing minimal drug to minimize potential side effects on the body and surrounding healthy tissue. Once the carrier accumulates in the chronic wound region via circulation or local application, the carrier material can precisely detect and respond to its unique microenvironmental cues, undergoing structural transformations (e.g., swelling, depolymerization, or chemical bond cleavage) to achieve controlled drug release at the most critical time and location [107]. This “sense–respond–release” paradigm greatly enhances the targeting, efficacy, and safety of treatment, representing an important evolution in wound repair from macroscopic dressings to micro-level intelligent therapy. Based on the core trigger signals they exploit, these smart systems can be broadly classified into several types.

#### 3.5.1. pH-Responsive Drug Delivery Systems

Local infection or ischemia in wounds often leads to a characteristic acidic microenvironment. This is primarily caused by bacterial metabolism producing lactic acid, combined with insufficient local blood perfusion, resulting in a pH typically ranging from 5.5 to 6.5—a notable gradient compared to the physiological pH of surrounding healthy tissue (~7.4) [108]. pH-responsive carriers are designed to exploit this difference for targeted drug release. The underlying principle involves incorporating proton-sensitive chemical bonds (e.g., hydrazone and imine) or functional groups (e.g., carboxyl groups) into the carrier material. Under acidic conditions, these structures undergo charge reversal, material swelling, or bond cleavage, thereby controlling the release of the loaded drug [109]. For example, PLGA-based nanoparticles loaded with baicalin contain ester bonds that hydrolyze more rapidly in acidic environments, leading to nanoparticle disintegration and rapid release of the antibacterial agent at the infection site [110]. Similarly, chitosan-based nanoparticles (with a pKa of ~6.5) experience increased protonation of amino groups in acidic wound environments, which promotes particle dissolution and accelerates the release of encapsulated drugs [111].

#### 3.5.2. Enzyme-Responsive Drug Delivery Systems

Exudates from chronic wounds typically contain abnormally high levels of various proteases, such as matrix metalloproteinases (MMPs, particularly MMP-2 and MMP-9) and elastase. The overexpression and heightened activity of these enzymes exacerbate pathological degradation of the extracellular matrix, thereby hindering normal repair processes [102]. Enzyme-responsive delivery systems are designed to exploit this characteristic, typically by linking therapeutic agents to the carrier via a peptide sequence specifically recognized and cleaved by the target protease (e.g., the sequence GPLGIAGQ), or by incorporating such peptides as “gatekeeping” structures within the carrier. Upon reaching wound regions enriched in the target proteases, these peptide “locks” are cleaved, enabling precise local release of the drug at the pathological site [112]. For instance, a study developed a hyaluronic acid hydrogel crosslinked with MMP-9-cleavable peptides to carry VEGF. This design allowed VEGF to be predominantly released in regions where the hydrogel was degraded by MMP-9, thereby achieving targeted pro-angiogenic activity at sites with the highest protease activity, which are typically the area most in need of repair [113].

#### 3.5.3. Glucose-Responsive Drug Delivery Systems

This system primarily targets the persistently hyperglycemic microenvironment characteristic of diabetic wounds. The core design strategy utilizes glucose itself as a trigger signal, converting fluctuations in glucose concentration into changes in the physicochemical properties of the carrier, thereby enabling on-demand drug release. Currently, two main mechanisms have been widely studied and applied [114].

The first mechanism is based on cascade catalytic reactions mediated by glucose oxidase (GOx). GOx catalyzes the oxidation of glucose in wound exudate to produce gluconic acid and H_2_O_2_. This reaction has a dual effect: on one hand, the accumulation of gluconic acid lowers the local pH; on the other hand, the generated H_2_O_2_ significantly increases ROS levels. Therefore, a delivery system integrated with GOx effectively amplifies the hyperglycemic signal and converts it into stronger pH reduction and oxidative stress signals, thereby synergistically triggering preprogrammed pH- or ROS-responsive release mechanisms within the carrier [114]. For example, a study co-encapsulated GOx and insulin into H_2_O_2_-sensitive nanomicelles; in a high-glucose environment, H_2_O_2_ was spontaneously generated, triggering micelle disassembly. This mimicked the physiological insulin secretion pattern, providing a smart strategy for managing hyperglycemia and promoting healing in diabetic wounds [115].

The second mechanism relies on the reversible covalent binding of phenylboronic acid (PBA) derivatives. PBA groups can reversibly form cyclic boronate esters with cis-diol structures in glucose molecules under alkaline or physiological pH conditions. This interaction alters the hydrophilicity/hydrophobicity or crosslinking state of PBA-containing polymers or nanocarriers, leading to swelling, disassembly, or degradation, thereby releasing the loaded drug [116]. Unlike the “active catalytic amplification” of GOx, PBA-based systems act more like a “dynamic equilibrium switch,” with response speed and glucose sensitivity tunable through chemical modification. Studies have demonstrated that microneedle patches or hydrogels based on PBA–glucose interactions can achieve glucose-dependent sustained release of growth factors (e.g., VEGF) at diabetic wound sites, effectively promoting angiogenesis [117].

Recent trends focus on integrating multiple mechanisms to enhance performance. For instance, combining GOx with PBA materials allows the acid generated by GOx to optimize the environment for PBA–glucose binding, resulting in a faster and more complete drug release system [118]. Such smart systems open promising avenues for precise, on-demand delivery of insulin, growth factors, or other therapeutics in diabetic wounds. However, their long-term biocompatibility, enzyme stability, and reliability in complex wound exudates remain critical challenges to address before clinical translation.

#### 3.5.4. ROS-Responsive Drug Delivery Systems

A major factor contributing to impaired healing in chronic wounds is the sustained presence of high levels of reactive oxygen ROS, such as H_2_O_2_ and ·OH. This oxidative stress directly damages cells and impedes the tissue repair process. To address this challenge, researchers have developed intelligent drug delivery systems capable of “sensing” and responding to ROS. The key design principle involves incorporating ROS-sensitive chemical moieties into the carrier materials, such as thioether bonds, diselenide bonds, arylboronate esters, or thioketal linkages. When these drug-loaded systems reach ROS-rich wound sites, these chemically labile groups act as “sensitive switches,” undergoing oxidation and thereby inducing significant changes in the material’s properties—for instance, converting from hydrophobic to hydrophilic or triggering cleavage of specific chemical bonds. As a result, the encapsulated drugs are released in a targeted manner. Notably, the oxidation process itself consumes part of the ROS, simultaneously alleviating local oxidative stress while facilitating site-specific drug delivery [119].

Based on this ingenious principle, a variety of formulations and applications have been developed. A typical example involves the use of thioether-containing polymers to construct micelles for curcumin delivery. In the ROS-rich environment of the wound, the thioether bonds are oxidized to more hydrophilic sulfoxides or sulfones, leading to micelle disassembly and rapid release of curcumin. This design achieves a dual benefit: it delivers the anti-inflammatory drug precisely while simultaneously consuming part of the ROS through the chemical reaction. Animal studies have demonstrated that this system promotes wound healing more effectively than curcumin alone. Beyond micelles, this strategy has been extended to other carrier platforms. For instance, arylboronate esters have been employed as linkers to construct ROS-responsive prodrugs, and selenium-containing amphiphilic molecules have been used to self-assemble into nanoparticles. These systems can intelligently respond to ROS levels at the wound site and have been successfully applied to deliver various antioxidant-active TCM components, such as tanshinone and baicalin [120].

#### 3.5.5. Thermoresponsive Drug Delivery Systems

During inflammation, the local temperature of a wound typically rises slightly, about 1–3 °C above normal body temperature, due to increased metabolism and blood flow [121]. Thermoresponsive drug delivery systems cleverly exploit this physiologically generated signal as a trigger. The key feature of these systems lies in the use of temperature-sensitive materials with a defined temperature threshold—the lower critical solution temperature (LCST). Among these, poly(N-isopropylacrylamide) (PNIPAM) and its derivatives have been the most extensively studied. These materials exhibit an interesting property: below the threshold, their polymer chains are hydrophilic and extended, and the system behaves like a liquid; once the temperature reaches or exceeds the threshold, the chains become hydrophobic and collapse, leading to disassembly of nanoparticles or instantaneous gelation of a liquid precursor [122,123].

This characteristic provides practical value. When drug-loaded thermoresponsive nanoparticles or solutions are applied to a wound, they remain stable at normal body temperature. Upon reaching the inflamed core region with slightly elevated temperature, the material undergoes a phase transition. This change can directly trigger rapid drug release or convert a liquid precursor into a solid gel that adheres to the wound. The resulting gel not only allows sustained and controlled drug release but also acts as a physical barrier to maintain a moist healing environment [124]. For example, in one study aimed at improving conventional artificial lenses, a Pluronic-based thermosensitive composite hydrogel was developed, integrating antibacterial, anti-inflammatory, and photothermal functionalities to create an injectable system that solidifies in situ with tunable refractive properties. The design is ingenious: after cataract surgery, the liquid hydrogel can be injected into the capsular bag, thermally gelled under near-infrared irradiation, and then rapidly cured with blue light into a complete lens. This system not only exhibits excellent optical performance and adjustable refractive power but also allows the encapsulated antibacterial and anti-inflammatory agents to act locally within the eye. Such a strategy combining “temperature sensing—state change—therapeutic release” achieves automatic targeting of inflamed regions and controlled drug delivery [124].

#### 3.5.6. Externally Stimuli-Responsive Systems

Unlike systems that rely on endogenous signals, externally stimuli-responsive systems use applied physical fields—such as NIR light, ultrasound, or alternating magnetic fields—to achieve remote, real-time, and high spatiotemporal precision in drug release [125,126].

The response mechanisms depend on the integration of specialized functional materials within the carrier. NIR-responsive systems typically incorporate photothermal agents, such as gold nanoshells/nanorods, black phosphorus quantum dots, or polydopamine nanoparticles. NIR light penetrates tissue efficiently, and upon irradiation, these photothermal agents convert light energy into heat, inducing local temperature rises that trigger phase transitions or chemical bond cleavage in the carrier (e.g., thermosensitive liposomes and polymers), resulting in drug release [127]. Magnetic-responsive systems usually employ superparamagnetic nanoparticles, such as Fe_3_O_4_. Under an applied alternating magnetic field, these particles generate magnetic hysteresis heating, which can similarly trigger thermally responsive drug release, or a static magnetic field gradient can be used to physically guide and localize the drug-loaded particles [128].

These systems offer significant practical advantages. First, they allow precise spatiotemporal control, enabling clinicians to deliver drugs in single or multiple pulses to specific wound regions (e.g., deep infection sites), and maximizing local efficacy while minimizing systemic exposure [129]. Second, they enable synergistic therapeutic modalities: the photothermal or magnetic heating itself can kill bacteria (including drug-resistant strains), while the released drugs (e.g., antibiotics and antioxidants) act via biochemical pathways. For instance, one study constructed hollow mesoporous silica nanoparticles coated with polydopamine and loaded with antibiotics. Upon NIR irradiation, the photothermal effect of polydopamine both promoted antibiotic release and directly contributed to bacterial killing, demonstrating an enhanced “thermotherapy + chemotherapy” effect against biofilm infections [130]. Although most of these systems remain in preclinical development and require specialized equipment, they represent a cutting-edge direction toward noninvasive, programmable, and personalized wound therapies.

#### 3.5.7. Multi-Responsive and Cascade-Responsive Systems

**Figure 4:**
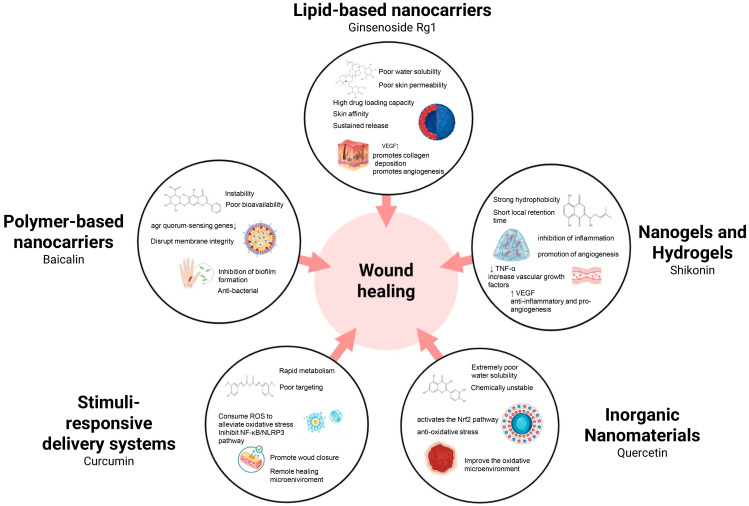
Schematic representation of major nanocarrier platforms and representative TCM-derived bioactive compounds used in wound healing. ↓ (Downward Arrow, next to TNF-α): Represents a decrease or downregulation of Tumor Necrosis Factor-alpha (TNF-α), a pro-inflammatory cytokine; ↑ (Upward Arrow, next to VEGF): Represents an increase or upregulation of Vascular Endothelial Growth Factor (VEGF), a key mediator of angiogenesis.

Wound healing is a dynamically evolving process, with the microenvironment exhibiting complex combinations and fluctuations of signals across both time and space. Single-responsive systems (e.g., those sensitive to only pH) can provide basic targeting but may lack sufficient specificity, making it difficult to accurately differentiate pathological regions at different healing stages or with overlapping characteristics. Consequently, recent research has focused on designing intelligent systems capable of responding to two or more endogenous or exogenous signals simultaneously, thereby greatly enhancing the precision and specificity of drug release [76]. Given the complexity of the wound microenvironment, a single signal may not offer enough specificity. The emerging trend is to develop systems responsive to multiple signals to improve targeting accuracy. For instance, a nanoparticle incorporating both pH-sensitive and ROS-sensitive linkages can achieve faster and more complete drug release in infected wounds (acidic and high ROS) while remaining relatively stable in microenvironments exhibiting only one of these characteristics [131]. Other systems may employ cascade reactions. For example, glucose oxidase reactions first generate H_2_O_2_ and provide an acidic microenvironment, which then amplify and trigger subsequent ROS- or pH-sensitive responses, enabling signal amplification and precise control. An overview of the different nanocarrier systems discussed in this section, along with typical TCM-derived compounds delivered by each platform, is illustrated in Figure 4.

Stimuli-responsive and smart delivery systems provide an attractive route to align on-demand release with dynamic wound microenvironments, enabling multifunctional interventions for chronic and diabetic wounds. However, a central translational barrier is the pronounced temporal and spatial heterogeneity of these cues across patients, wound types, and healing stages, which makes trigger thresholds and response kinetics difficult to standardize and may reduce the predictability of simplified in vitro testing [132,133,134]. In addition, increasing system complexity can improve proof-of-concept performance but often introduces challenges in reproducible manufacturing, definition of critical quality attributes, long-term stability, and practical clinical workflows (e.g., equipment requirements, penetration depth, dosing control, and cost) [132,135,136]. Therefore, future designs should emphasize “translatable smartness”: selecting clinically robust triggers, simplifying architectures when possible, validating performance under wound-relevant conditions, and explicitly addressing manufacturability, quality control, and usability early in development. The design strategies, triggering signals, and carrier materials of stimuli-responsive smart delivery systems for TCM compounds in wound healing are outlined in Table 4.

## 4. Challenges and Perspectives

Despite the encouraging progress summarized above, it should be noted that many current studies remain at the proof-of-concept stage. Most nanocarrier systems have been evaluated primarily in simplified in vitro models or small animal wound models, which may not fully recapitulate the complexity of human chronic wounds. The pathological microenvironment of clinical wounds is highly heterogeneous and dynamic, characterized by fluctuating bacterial burden, protease activity, oxygen levels, and immune responses. These factors can significantly influence the stability, degradation, and drug-release behavior of nanocarriers in vivo. As a result, therapeutic performance observed in laboratory settings may not directly translate to clinical outcomes.

**Figure 5:**
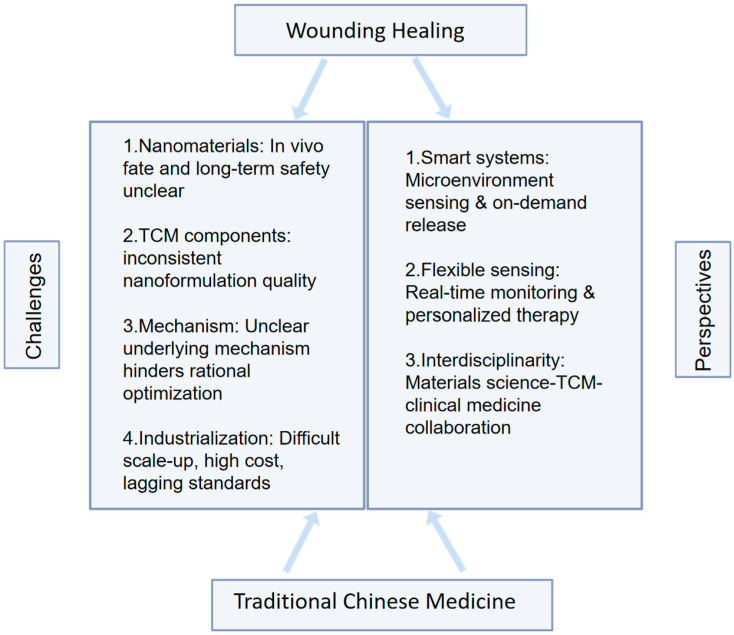
Challenges and perspectives in the clinical application of Traditional Chinese Medicine (TCM) [138].

In addition, although multifunctional nanoplatforms often demonstrate enhanced therapeutic efficacy in experimental models, increasing system complexity can introduce new challenges related to reproducibility, manufacturing consistency, and regulatory evaluation. From a translational perspective, the balance between functional sophistication and practical manufacturability remains an important consideration for the development of clinically viable nanomedicines for wound therapy. Key therapeutic outcomes reported in preclinical and clinical studies using TCM-derived compounds loaded in nanocarriers are summarized in Table 5. Although the integration of bioactive compounds from TCM with nanodelivery technologies offers novel strategies for wound healing, several critical challenges remain before clinical translation. First, the in vivo fate and long-term safety of nanomaterials are still insufficiently characterized, especially for systems involving inorganic components or highly engineered multi-functional architectures, which may raise concerns regarding chronic exposure, persistence, and benefit–risk balance. Second, the intrinsic variability of TCM-derived ingredients introduces an additional layer of complexity in achieving consistent quality and reproducible therapeutic performance. Third, the interactions among “TCM component–nanocarrier–wound microenvironment” often remain incompletely understood, limiting rational optimization and making it difficult to establish robust structure–activity relationships. Furthermore, scaling up from laboratory research to industrial production faces practical bottlenecks, including process robustness, cost, and the lack of standardized evaluation protocols under clinically relevant wound conditions (Figure 5).

From a translational and pharmaceutics perspective, regulatory expectations and product positioning should be considered early, because the likely regulatory pathway directly shapes CMC requirements, safety assessment depth, and evidence expectations. Depending on the intended clinical use, TCM-assisted nanoformulations for wound care may be developed under a botanical-drug-like paradigm, as nanotechnology-involving drug products, or as dressing-based combination products where the matrix contributes materially to performance. Different pathways place different emphasis on raw material control, nanoscale characterization, manufacturing changes, and product specifications, and therefore require a clear definition of the “active”, the key claims, and the critical quality attributes linked to safety and efficacy [137,138,139].

Another major barrier is the reproducibility of TCM-derived inputs. Variations in herbal sources, processing, and extraction conditions can substantially alter chemical profiles, leading to batch-to-batch differences in pharmacological activity and formulation behavior. For translation, a practical standardization framework is needed beyond proof-of-concept demonstrations. This includes well-defined raw-material specifications, marker-based quantification combined with holistic fingerprinting where appropriate, and batch-release criteria explicitly linked to biological activity and formulation performance [137,138].

In addition, manufacturing scalability and industrial feasibility remain under-addressed in many academic studies. While complex smart systems and multi-responsive designs can improve precision in preclinical settings, they often increase process complexity, hinder scale-up, and complicate quality control. Industrially relevant considerations—including scalable preparation methods, sterilization compatibility, storage stability, packaging, cost-effectiveness, and reproducible performance in exudate-/biofilm-relevant conditions—should be incorporated as early design constraints rather than treated as post hoc issues. A quality-by-design mindset can help bridge the gap from laboratory batches to reproducible products suitable for clinical development [140,141].

Only through such cross-disciplinary synergy—grounded in clarifying safety profiles and mechanisms—can this strategy, which merges traditional wisdom with cutting-edge technology, move beyond the laboratory and provide tangible improvements in the management of complex wounds.

From a future development perspective, greater attention should also be paid to regulatory pathways and industrial translation. The successful clinical application of nanotechnology-enabled TCM formulations will require standardized characterization of nanocarriers, well-defined quality attributes, and reproducible manufacturing processes that comply with regulatory requirements. In addition, large-scale production methods that ensure batch-to-batch consistency, stability during storage, and compatibility with sterilization procedures will be essential for commercialization. Close collaboration among materials scientists, pharmacologists, clinicians, and regulatory experts will therefore be necessary to bridge the gap between laboratory innovation and clinically deployable wound therapies.

## 5. Conclusions

Bioactive compounds derived from TCM exert important therapeutic effects during wound healing through multitarget and multipathway regulation, exhibiting synergistic actions particularly in key processes such as antimicrobial defense, inflammation modulation, oxidative stress attenuation, angiogenesis, and collagen remodeling. However, many of these compounds suffer from intrinsic limitations, including poor aqueous solubility, low chemical stability, limited bioavailability, and insufficient ability to penetrate the skin barrier, which substantially restrict their practical clinical application.

The emergence of nanodelivery technologies has provided new avenues to address these challenges. By employing carriers such as liposomes, polymeric nanoparticles, and nanogels, the solubility and stability of TCM bioactive compounds can be markedly improved, while their local retention and penetration at wound sites are enhanced. Moreover, stimulus-responsive nanocarriers enable drug release to be regulated by changes in the wound microenvironment, allowing therapeutic interventions to become more precise and controllable.

Although the strategy combining TCM bioactive compounds with nanodelivery systems has demonstrated considerable promise in enhancing antimicrobial efficacy, modulating immune responses, alleviating oxidative stress, and accelerating wound closure, significant obstacles remain before clinical translation can be realized. These include the need for systematic evaluation of long-term nanomaterial safety, challenges in ensuring formulation consistency due to variability in herbal sources, incomplete understanding of the mechanisms underlying complex composite systems, and technical barriers associated with scaling up from laboratory preparation to industrial production. Therefore, continued efforts in safety assessment, quality control, mechanistic elucidation, and process translation are essential for this integrated strategy to ultimately evolve into a reliable and personalized therapeutic option for clinical wound management.

Overall, nanotechnology-assisted delivery systems provide several key advantages for the therapeutic application of TCM-derived compounds. These systems can enhance drug solubility and stability, improve penetration and retention at wound sites, enable controlled and stimuli-responsive drug release, and allow the integration of multiple therapeutic functions within a single platform. By combining the multi-target pharmacological properties of TCM with the precise delivery capabilities of nanotechnology, these strategies hold considerable promise for improving the treatment of complex and chronic wounds.

## Figures and Tables

**Table 1:** Major TCM-derived natural compounds discussed in this review: chemical structures and botanical sources.

Compound	TCM Source	Chemical Structures
Baicalin	*Scutellaria baicalensis*	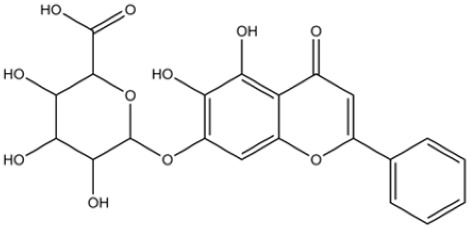
Emodin	*Rheum palmatum*	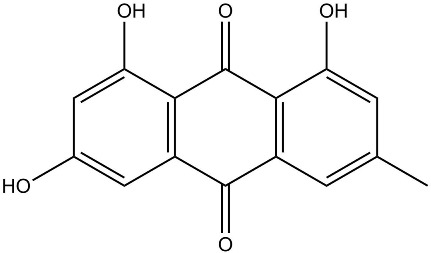
Berberine	*Coptis chinensis*	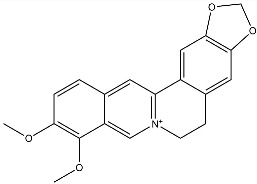
Paeonol	*Paeonia suffruticosa*	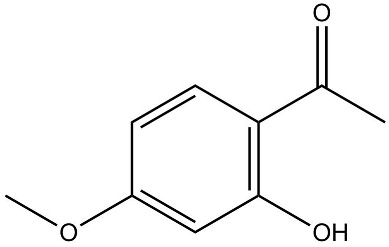
Ginsenoside Rg1	*Panax ginseng*	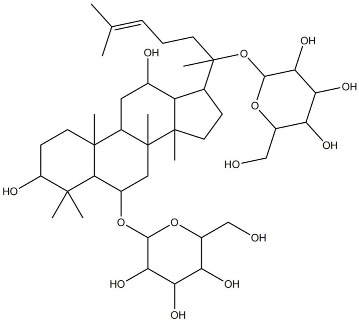
Astragaloside IV	*Astragalus membranaceus*	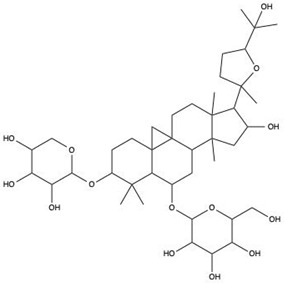
Curcumin	*Curcuma longa*	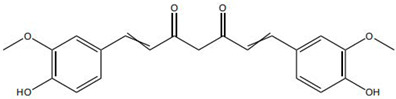
Tanshinone IIA	*Salvia miltiorrhiza*	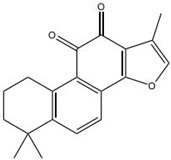
Asiaticoside	*Centella asiatica*	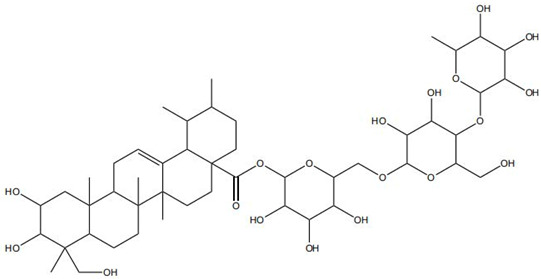
Salidroside	*Rhodiola rosea*	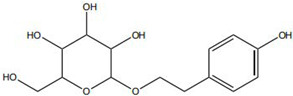
Icariin	*Epimedium brevicornu*	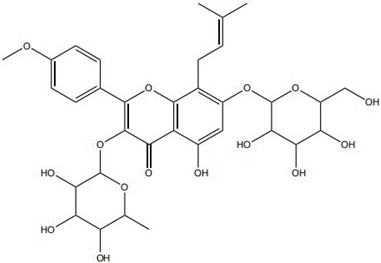
Xanthotoxin	Multiple TCM plant sources (representative: *Angelica dahurica*)	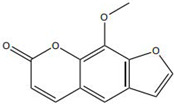
Quercetin	Multiple TCM plant sources (representative: *Sophora japonica*)	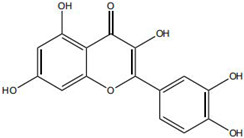
Gallic acid	Multiple TCM plant sources (representative: *Rhus chinensis* Mill.)	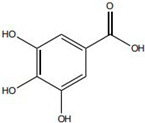
Coptisine	*Coptis chinensis*	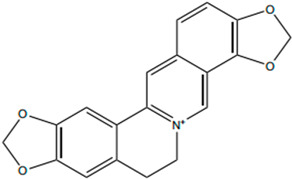
Celastrol	*Tripterygium wilfordii*	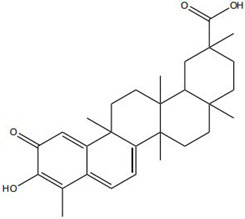
Resveratrol	*Polygonum cuspidatum* (common source)	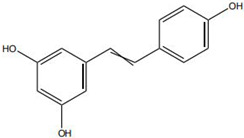
Rutin	Multiple TCM plant sources (representative: *Sophora japonica*)	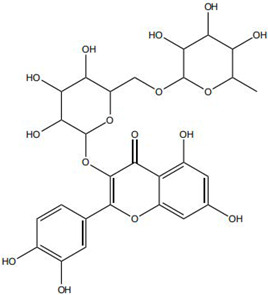
Luteolin	Multiple TCM plant sources (representative: *Lonicera japonica*)	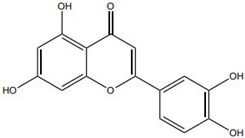
Apigenin	Multiple TCM plant sources (representative: *Chrysanthemum morifolium*)	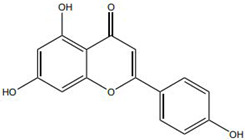
Ginsenoside Rb1	*Panax ginseng*	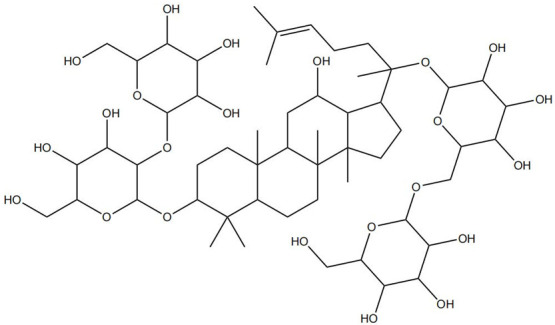
Ferulic acid	*Angelica sinensis* (common source)	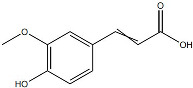
Notoginsenoside R1	*Panax notoginseng*	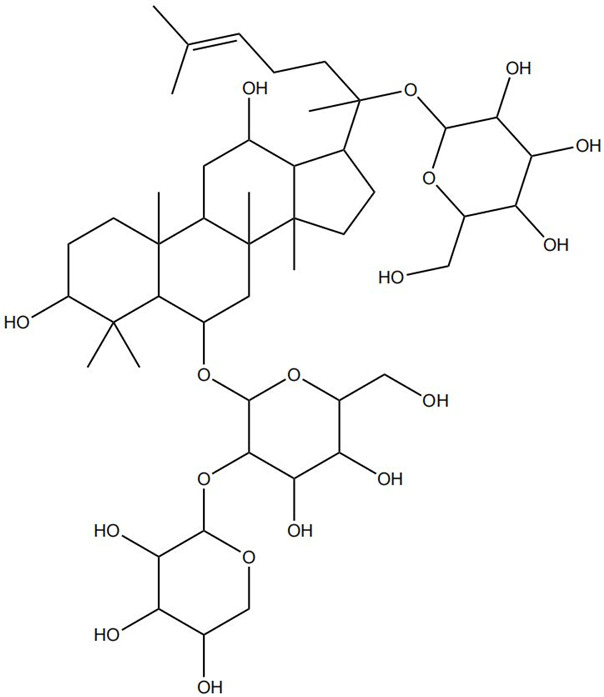
Ginsenoside Rg3	*Panax ginseng*	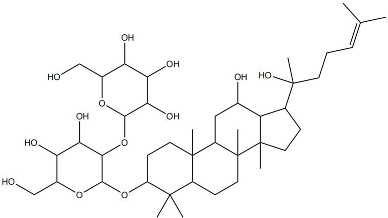
Shikonin	*Lithospermum erythrorhizon*	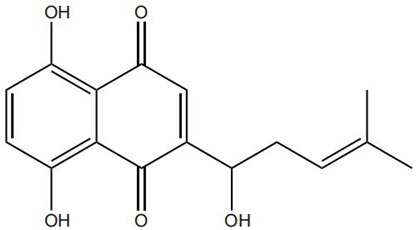
Panax notoginseng saponins	*Panax notoginseng*	Mixture (standardized extract; major components include notoginsenoside R1, ginsenoside Rg1, Rb1, etc.)

**Table 2:** Comparison of biological mechanisms of compounds derived from Traditional Chinese Medicine.

Compound	TCM Source	Primary Biological Mechanism	Advantages	Limitations
Baicalin	*Scutellaria baicalensis*	Inhibits bacterial quorum sensing, downregulates agr gene expression	Disrupts biofilms, enhances antibiotic penetration	Less effective against Gram-negative bacteria
Emodin	*Rheum palmatum*	Downregulates icaA/sarA genes, inhibits PIA synthesis	Targets key biofilm formation genes	Slow action, requires sustained exposure
Berberine	*Coptis chinensis*	Disrupts bacterial membrane integrity, inhibits efflux pumps	Overcomes drug resistance, synergizes with antibiotics	Poor water solubility, low bioavailability
Paeonol	*Paeonia suffruticosa*	Inhibits bacterial adhesion, disrupts extracellular polysaccharide	Concurrent anti-inflammatory effects	Poor stability
Ginsenoside Rg1	*Panax ginseng*	Activates PI3K/Akt pathway, promotes M2 polarization	Dual immunomodulatory and pro-proliferative effects	Low oral bioavailability
Astragaloside IV	*Astragalus membranaceus*	Activates STAT6 pathway, upregulates Arg-1 and IL-10	Potently promotes M2 polarization	Complex extraction process
Curcumin	*Curcuma longa*	Inhibits NF-κB and NLRP3 inflammasome	Multi-target anti-inflammatory, high safety	Poor photostability, rapid metabolism
Tanshinone IIA	*Salvia miltiorrhiza*	Activates Nrf2 pathway, upregulates antioxidant enzymes	Sustained antioxidant effects, enhances endogenous defense	Extremely poor water solubility
Asiaticoside	*Centella asiatica*	Upregulates TGF-β1, modulates MMP/TIMP balance	Promotes ordered collagen deposition	May inhibit cell proliferation at high concentrations
Salidroside	*Rhodiola rosea*	Upregulates VEGF/bFGF expression	Potently promotes angiogenesis	Short in vivo half-life
Icariin	*Epimedium brevicornu*	Activates Src/PI3K/Akt pathway	Dual angiogenic and osteogenic effects	Studies mostly focused on bone tissue
Xanthotoxin	Various TCM herbs	Stabilizes HIF-1α, activates downstream pro-angiogenic genes	Responds to hypoxic microenvironment, physiological regulation	Potential phototoxicity risk

**Table 3:** Comparison of physicochemical and functional characteristics of different nanocarriers.

Carrier Type	Structural Characteristics	Advantages	Active Ingredients	Mechanism	Therapeutic Benefit	Experimental Model	Key Quantitative Results	Ref.
Liposomes	Phospholipid bilayer vesicles with aqueous core and hydrophobic bilayer	High biocompatibility, mimics cell membrane, surface modifiable	Shikonin	Enhanced transdermal delivery, sustained release	Anti-inflammatory, pro-angiogenic, scar prevention	Deep second-degree burn mouse model	Wound closure rate at day 14: 92.3% vs. 68.7% (control); scar width reduced by 41.2%	[62]
Solid Lipid Nanoparticles (SLN)	Solid lipid core (solid at physiological temperature)	Good physical stability, controlled release	Ginsenoside Rg3	Sustained release, improved stability	Anti-inflammatory, promotes granulation tissue formation	Full-thickness wound mouse model	Wound closure at day 14: 95.1% vs. 72.4% (control); CD31+ area increased by 62%	[59]
Nanostructured Lipid Carriers (NLC)	Solid + liquid lipid mixture, imperfect crystal structure	High drug loading, good stability, enhanced transdermal	Curcumin, resveratrol, ginsenoside Rg1	High encapsulation efficiency, prolonged skin retention	Antioxidant, anti-inflammatory, promotes re-epithelialization	Ex vivo skin deposition model	Curcumin skin deposition increased by 2.3-fold vs. free curcumin; permeation flux 12.6 µg/cm^2^/h	[60]
PLGA Nanoparticles	Biodegradable synthetic polymer	FDA-approved, tunable degradation, long-term release	EGF, bFGF, baicalin	Controlled release, protection from enzymatic degradation	Promotes angiogenesis, cell proliferation, ECM synthesis	In vitro cell migration assay	EGF-loaded PLGA NPs significantly promoted fibroblast migration (scratch assay, closure rate 82% at 24 h vs. 45% control)	[62]
Chitosan Nanoparticles	Cationic polysaccharide, ionically crosslinked	Intrinsic antibacterial, mucoadhesive, biocompatible	Baicalin, protein-based therapeutics	pH-responsive swelling, electrostatic interaction with bacteria	Antibiofilm, enhances drug retention, promotes healing	In vitro antibacterial and cell migration assays	MIC against *S. aureus* 2 mg/mL; cell proliferation rate 616% over 7 days; particle size 458.39 nm	[64]
Hyaluronic Acid (HA) Nanoparticles	Natural polysaccharide, self-assembled or crosslinked	Targets CD44+ cells, modulates inflammation and hydration	Anti-inflammatory drugs (e.g., curcumin analogs)	Receptor-mediated targeting, immune modulation	Targeted delivery, promotes M2 macrophage polarization	Human ex vivo wound model	Cellular uptake reached 80% within 4 h; significantly increased expression of keratin 17 and CD31 (*p* < 0.0001), promoting re-epithelialization and angiogenesis	[66]
Mesoporous Silica Nanoparticles (MSN)	Highly ordered nanochannels, large surface area	Ultra-high loading, easy surface modification, responsive design	Quercetin, tanshinone IIA	pH/ROS-responsive release, high drug loading	Antioxidant, anti-inflammatory, pro-angiogenic	Diabetic wound mouse model	Wound closure rate: 85% at day 14 vs. 60% control; promoted collagen deposition and angiogenesis	[78]
Metal Nanoparticles (Ag, ZnO)	Metal/metal oxide core	Intrinsic antibacterial, pro-healing (Zn^2+^)	Gallic acid (with ZnO)	Ion release, ROS generation, membrane disruption	Synergistic antibacterial, promotes re-epithelialization	In vitro antibacterial and cytotoxicity assays	MIC 0.625 mg/mL; significant inhibition of biofilm formation; cell viability 99.07%	[80]
Nanozymes (CeO_2_, Fe_3_O_4_)	Inorganic nanoparticles with enzyme-like activity	Sustained ROS scavenging, stable microenvironment response	– (carrier + therapeutic)	SOD/catalase-mimetic activity, macrophage modulation	Reduces oxidative stress, promotes M2 polarization	Diabetic infected wound mouse model	Accelerated wound healing (closure rate approx. 82% at day 14 vs. 60% control); promoted collagen deposition and epithelial regeneration	[85]
Photothermal-Responsive Nanoparticles (Au, polydopamine)	Core–shell structures with photothermal conversion capacity	Remote control, spatiotemporal precision, synergistic hyperthermia	Antibiotics (e.g., in Au@MSN)	NIR-triggered drug release, photothermal biofilm disruption	Enhanced antibiofilm, synergistic chemo-photothermal therapy	Photothermal performance characterization (in vitro)	TaS_2_ nanoparticles exhibited excellent photothermal conversion efficiency in NIR-II region (1000–1700 nm); optimal performance with small size (<50 nm)	[87]

**Table 4:** Comparison of stimuli-responsive smart delivery systems for TCM compounds in wound healing.

Stimulus Type	Response Mechanism	Carrier Material/Polymer
pH-Responsive	Protonation, charge reversal, bond cleavage (hydrazone, imine, ester)	PLGA, Chitosan
Enzyme-Responsive	Peptide cleavage by proteases (MMP-2/9, elastase)	Hyaluronic acid hydrogels with MMP-cleavable peptides
Glucose-Responsive (GOx-based)	Glucose oxidase converts glucose to gluconic acid + H_2_O_2_; triggers pH/ROS-responsive release	H_2_O_2_-sensitive nanomicelles
Glucose-Responsive (PBA-based)	Reversible covalent binding between PBA and glucose; alters polymer hydrophilicity/crosslinking	PBA-containing polymers, hydrogels, microneedles
ROS-Responsive	Oxidation of sensitive moieties (thioether, diselenide, arylboronate, thioketal); converts hydrophobic to hydrophilic or cleaves bonds	Thioether-containing polymers, arylboronate-linked prodrugs, selenium-containing amphiphiles
Thermoresponsive	Phase transition at LCST; polymer chains collapse at elevated temperature	PNIPAM and derivatives, Pluronic-based hydrogels
Externally Triggered (NIR)	Photothermal conversion; heat triggers phase transition or bond cleavage	Gold nanoshells/nanorods, black phosphorus, polydopamine, Au@mesoporous SiO_2_
Externally Triggered (Magnetic)	Magnetic hysteresis heating or field-guided localization	Superparamagnetic iron oxide (Fe_3_O_4_)
Multi-Responsive (pH + ROS)	Combined pH-sensitive and ROS-sensitive linkages	Nanoparticles with dual-sensitive bonds
Cascade-Responsive	Sequential signal amplification (e.g., GOx generates H_2_O_2_ and acid, triggering ROS/pH response)	Integrated GOx with ROS/pH-sensitive carriers

**Table 5:** Comparison of therapeutic outcomes reported in preclinical/clinical studies.

TCM Compound	Nanocarrier	Experimental Model	Primary Efficacy
Ginsenoside Rg1	NLC + CMC gel	Diabetic rat full-thickness skin defect	Promotes collagen deposition, angiogenesis, re-epithelialization
Ginsenoside Rg3	SLN gel	Mouse full-thickness skin defect	Anti-inflammatory, promotes granulation tissue
Curcumin	NLC	Ex vivo skin permeation model	Enhanced skin retention, sustained release
Shikonin	Liposomes + thermosensitive chitosan hydrogel	Rat deep second-degree burn	Anti-inflammatory, pro-angiogenic, scar inhibition
Quercetin	Mesoporous silica nanoparticles	Diabetic wound model	Antioxidant, anti-inflammatory, pro-angiogenic
Baicalin	pH-responsive PLGA nanoparticles	In vitro release/bacterial infection model	Site-specific release at infection site
Curcumin	ROS-responsive thioether micelles	Oxidative stress model	ROS-triggered release, concurrent ROS scavenging

## Data Availability

No new data were created or analyzed in this study.

## Abbreviations

TCM	Traditional Chinese Medicine
ROS	Reactive oxygen species
ECM	Extracellular matrix
PIA	Polysaccharide intercellular adhesin
TNF-α	Tumor necrosis factor-α
IL-1β	Interleukin-1β
IL-10	Interleukin-10
TGF-β	Transforming growth factor-β
Arg-1	Arginase-1
O_2_^−^	Superoxide anions
·OH	Hydroxyl radicals
Nrf2	Nuclear factor erythroid 2-related factor 2
SOD	Superoxide dismutase
GPx	Glutathione peroxidase
AGEs	Advanced glycation end products
RAGE	Advanced glycation end product receptor
VEGF	Vascular endothelial growth factor
bFGF	Basic fibroblast growth factor
HIF-1α	Hypoxia-inducible factor-1α
PEG	Polyethylene glycol
SLNs	Solid lipid nanoparticles
NLCs	Nanostructured lipid carriers
PLGA	Poly(lactic-co-glycolic acid)
EGF	Epidermal growth factor
STPP	Sodium tripolyphosphate
HA	Hyaluronic acid
MSNs	Mesoporous silica nanoparticles
CeO_2_	Cerium oxide
Fe_3_O_4_	Magnetite
CAT	Catalase
H_2_O_2_	Hydrogen peroxide
NIR	Near-infrared
MMPs	Matrix metalloproteinases
GOx	Oxidase
PBA	Phenylboronic acid
LCST	Lower critical solution temperature
PNIPAM	Poly(N-isopropylacrylamide)
